# Gonadotropin levels at the start of ovarian stimulation predict normal fertilization after hCG re‐trigger in GnRH antagonist cycles

**DOI:** 10.1002/rmb2.12359

**Published:** 2020-12-18

**Authors:** Hiroya Kitasaka, Mikiko Tokoro, Masae Kojima, Noritaka Fukunaga, Yoshimasa Asada

**Affiliations:** ^1^ Asada Ladies Clinic Nagoya Japan; ^2^ Asada Institute for Reproductive Medicine Kasugai Japan

**Keywords:** controlled ovarian stimulation, gonadotropin‐releasing hormone antagonist protocol, gonadotropin‐releasing hormone agonist trigger, luteinizing hormone surge, oocyte maturation

## Abstract

**Purpose:**

To assess the appropriateness of human chorionic gonadotropin (hCG) re‐trigger in poor responders to gonadotropin‐releasing hormone agonist (GnRHa) trigger in controlled ovarian stimulation (COS) cycles.

**Methods:**

The 2251 cycles in 2251 patients triggered with GnRHa for oocyte stimulation, with or without requiring hCG re‐trigger between 2013 and 2018, were retrospectively analyzed to compare gonadotropin levels at the start of COS and the rate of normal fertilization between the re‐trigger and non–re‐trigger group. Furthermore, patients in the re‐trigger group were stratified by the rate of normal fertilization (good: ≥60% or poor: <60%) to compare patient demographics, hormone profiles, and clinical outcome between the subgroups.

**Results:**

In the re‐trigger group, FSH and LH levels at the start of COS were significantly lower in the good fertilization group than in the poor fertilization group (*P* < .01). Receiver operating characteristic curves identified cutoff values of the FSH and LH levels of 1.30 and 0.35 mIU/mL, respectively, for predicting ≥60% normal fertilization.

**Conclusion:**

Gonadotropin levels at the start of COS are predictors of response to GnRHa trigger and hCG re‐trigger necessity, and may serve as indicators to help clinicians appropriately choose hCG re‐trigger rather than abandoning the cycles or continuing the first oocyte aspiration attempt.

## Graphical abstract

When FSH and LH levels at the start of controlled ovarian stimulation were higher than the cutoff levels derived from ROC curve, oocytes obtained by re‐triggering with hCG frequently resulted in abnormal fertilization.

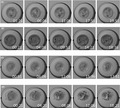

## 1 INTRODUCTION

Ovarian hyperstimulation syndrome (OHSS) is an iatrogenic complication of controlled ovarian stimulation (COS) protocols. The most common symptoms are decreased circulating blood volume^1^, ^2^ and pleural/peritoneal fluid accumulation,^3^ and in serious cases, resulting in fatal cardiorespiratory impairment. The most possible pathogenesis of OHSS is the increased release of vascular endothelial growth factor (VEGF) by human chorionic gonadotropin (hCG) administered as an ovulation trigger in COS protocols, which induces capillary hyperpermeability.^4^, ^5^, ^6^, ^7^ Although the reported incidence of OHSS in in vitro fertilization (IVF) cycles varies widely, mild OHSS and moderate‐to‐severe OHSS are expected to occur in 20%‐33% and 3%‐8%, respectively, of IVF cycles.^8^ In order to prevent OHSS, GnRH antagonist protocols using a GnRH agonist (GnRHa) as an ovulation trigger have been introduced in IVF cycles.^9^ GnRH antagonist protocols, as compared with conventional GnRHa long protocols, require a lower dose of gonadotropin medications^10^ and reduce the risk of serious OHSS.^11^ However, GnRHa trigger fails to achieve sufficient oocyte yields in some patients.^12^, ^13^, ^14^ Failures to retrieve adequate oocytes range from far fewer oocytes retrieved than expected from the number of mature follicles visible on the day of ovulation^15^ to empty follicle syndrome.^16^, ^17^

We have experienced such oocyte retrieval failures in patients undergoing GnRH antagonist protocols using GnRHa trigger. Some of the patients were rescued by re‐triggering oocyte maturation with hCG, leading to successful oocyte retrieval and resulting in live births.^18^, ^19^ However, the other patients were found to have morphologically abnormal cumulus‐oocyte complexes (COCs) and lower rates of normal fertilization after hCG re‐trigger. These experiences have led us to believe that hCG re‐trigger may not be appropriate for all poor responders to GnRHa trigger.

This study retrospectively assessed the appropriateness of conducting hCG re‐trigger based on fertilization outcomes and attempted to identify patient characteristics or hormone profiles, which might be predictive of normal fertilization after hCG re‐trigger.

## 2 MATERIALS AND METHODS

### 2.1 Statement of ethics

All the procedures accorded with the ethical standards of the relevant committees on human experimentation (institutional and national) and with the Helsinki Declaration of 1964 and its later amendments. The study design was approved by the ethics committee of the Institutional Review Board (IRB) of Asada Ladies Clinic. This is a retrospective study in patients who submitted informed consent for undergoing fertility treatment at our clinic. (IRB approval number: 2019‐10).

### 2.2 Patients

This study reviewed 2251 cycles of GnRH antagonist–based COS using GnRHa trigger for final oocyte maturation conducted in patients who received fertility treatment from 2013 to 2018 at our clinic. The patients who underwent oocyte retrieval more than once during the study period were not included. The opt‐out method (potential participants were informed about research, information was available on our Web site, and patients were included unless they objected) approved by the IRB of Asada Ladies Clinic was used for this study to recruit the participants.

Among these, 36 cycles, in which adequate oocytes could not be collected at the first oocyte aspiration attempt probably because of suboptimal oocyte maturation, required hCG re‐trigger; these cycles constituted the re‐trigger group. The remaining 2215 cycles requiring no hCG re‐trigger constituted the non‐re‐trigger group. Patients in the re‐trigger group were matched for age with those in the non–re‐trigger group to minimize age bias.

### 2.3 Treatment

#### 2.3.1 Ovarian stimulation and frozen‐thawed embryo transfer

Controlled ovarian stimulation was achieved with a flexible GnRH antagonist protocol. Patients received oral estrogen 2.0 mg (Julina tablets; Bayer Yakuhin) for 21 consecutive days from day 5 and oral progesterone 10.0 mg (Norluten; Fuji Pharma) for 10 days from day 16 in the preceding cycle.

On day 3 of the menstrual cycle, gonadotropin treatment started. The gonadotropin medication(s) used for ovarian stimulation was chosen based on LH activity at the start of COS: Human menopausal gonadotropin (hMG) (HMG for injection <FERRING>, Ferring Pharmaceuticals; or HMG injection Teizo; Aska Pharmaceutical) was used alone if the LH level was <2 mIU/mL, or in combination with recombinant FSH (follitropin alfa [Gonal‐F^®^]; Merck) if the LH level was ≥2 mIU/mL. Based on the facts that there is no report about LH surge occurring immediately after T_1/2_ and this protocol can reduce patient's burden (clinic visit time and cost), when the lead follicular size reached ≥16 mm in diameter, a GnRH antagonist (ganirelix [Ganirest^®^, 0.25 mg; MSD] or cetrorelix [Cetrotide^®^, 0.25 mg; Merck]) was initiated and continued every other day until the day final oocyte maturation was triggered. When three or more follicles reached a maximum diameter of 18‐20 mm, final oocyte maturation was triggered with a GnRHa, either leuprolide acetate administered as a single injection of 1 mg [Lucrin^®^ Injection; AbbVie] or buserelin acetate administered as four intranasal doses of 300 μg per both nostrils, that is, 1200 μg in total [BUSERECUR^®^; Fuji Pharma].

The GnRHa trigger was scheduled for 21:00 to 22:00 and oocyte retrieval was conducted 36‐40 hours after the GnRHa trigger. On the day of oocyte retrieval, 5‐10 follicles of ≥20 mm in diameter were initially aspirated from one or both ovaries. Patients with an oocyte recovery rate (the ratio of the number of collected oocytes to the number of aspirated follicles ≥20 mm in diameter) of >50% were deemed to require no re‐trigger and subsequent aspirations were continued (non–re‐trigger group). Patients with an oocyte recovery rate of ≤50%, and no or only a few oocytes were recovered, despite 5‐10 follicles measuring ≥20 mm in diameter aspirated, the first oocyte stimulation with GnRHa trigger was presumed to have been suboptimal and the patient was given an hCG injection for re‐triggering in the same cycle at the discretion of the clinicians (re‐trigger group). The other follicles were kept in ovaries for the second oocyte retrieval. The second oocyte retrieval was scheduled 36 hours after administration of 5000 IU (or 3333 IU if the risk of OHSS was expected to be increased) of hCG (human chorionic gonadotropin for injection; Fuji Pharma).

Retrieved oocytes were fertilized using intracytoplasmic sperm injection (ICSI), and the embryos were cultured for up to 20 hours in a time‐lapse imaging incubator (EmbryoScope; Vitrolife; or CCM‐iBIS NEXT; Astec) that acquired images at 15‐minute intervals. The appearance of two pronuclear (2PN) signified normal fertilization and that of ≥3 PN abnormal fertilization. All of the resultant embryos were cryopreserved at the pronuclear or blastocyst stage to prevent OHSS, until thawed for embryo transfer which was carried out in hormonally primed cycles using sequentially administered transdermal estradiol (Estrana^®^; Hisamitsu Pharmaceutical) and chlormadinone acetate (Lutoral^®^; Fuji Pharma).^20^ A clinical pregnancy was defined by the presence of a gestational sac by transvaginal ultrasound performed at approximately 4 weeks after embryo transfer.

### 2.4 Patient characteristics and hormone profiles

The following patient characteristics and hormone profiles were compared between the re‐trigger group and the non–re‐trigger group: age, anti‐Mullerian hormone (AMH) level, body mass index (BMI), total number of COS cycles, FSH and LH levels at the start of COS (day 3 of the menstrual cycle), total dose of GnRH antagonist required, total dose of gonadotropin required, serum estradiol (E2) and progesterone (P4) levels on the day of GnRHa trigger, and number of oocytes retrieved. We defined a 60% normal fertilization as a threshold for “good fertilization,” based on previously reported rate of normal fertilization in ICSI cycles (68.6%‐77.1%).^21^, ^22^, ^23^ The re‐trigger group consisting of 36 patients was subdivided into a group of a ≥60% normal fertilization (“the good fertilization group”) and a group of a <60% normal fertilization (“the poor fertilization group”) to assess the following patient characteristics and hormone profiles: age, AMH level, FSH and LH levels at the start of COS (day 3 of the menstrual cycle), serum E2 and P4 levels on the day of GnRHa trigger, P4 level on the day of hCG re‐trigger, and dose of hCG used for re‐trigger. In addition, clinical outcomes (clinical pregnancy rate, clinical loss rate, and live birth rate) were compared between the good fertilization group and the poor fertilization group.

### 2.5 Male partner's characteristics

To assess the effects of male partner's characteristics on fertilization outcomes, age, LH, FSH, total testosterone, semen volume, sperm concentration, and sperm motility in male partners were compared between the poor fertilization group and the good fertilization group.

### 2.6 Statistical analyses

Before between‐group comparisons, the Kolmogorov‐Smirnov test or the Shapiro‐Wilk test was conducted to assess data distribution in each group. For normally distributed data, *F* test was conducted and if the data showed homoscedasticity, *t* test was used to compare mean values between the groups. For data not normally distributed, the Mann‐Whitney *U* test was used to compare median values between the groups. Rates in the groups were compared with the use of Fisher's exact test. The proportion of patients using each GnRHa medication was compared using chi‐squared test. A free software “R” was used for all data analyses. A *P‐value* of <.05 was considered statistically significant.

## 3 RESULTS

### 3.1 Comparisons of patient characteristics and hormone profiles between the re‐trigger group and the non–re‐trigger group

There were no significant differences between the re‐trigger group and the non–re‐trigger group in age, AMH level, BMI, the total number of COS cycles, the total dose of GnRH antagonist required, and serum E2 and P4 levels on the day of GnRHa trigger (Table 1). In the re‐trigger group, the total dose of gonadotropin required was significantly higher, and FSH and LH levels at the start of COS were significantly lower than those in the non–re‐trigger group (all *P* < .01). Since a decision to re‐trigger was made by clinicians on the fact that no or only a few oocytes were retrieved, the number of oocytes retrieved in the re‐trigger group (0.5 oocytes) was significantly lower than that in the non–re‐trigger group (23 oocytes, *P* < .01).

**TABLE 1:** Characteristics and hormone profiles in patients treated with GnRHa trigger for final oocyte maturation (non–re‐trigger group versus re‐trigger group)

	Non–re‐trigger	Re‐trigger	*P*‐value^a^
Number of patients	2215	36	—
*N* (number of cycles)	2215	36	—
Age at the start of COS (Y)	34 (31‐37)	35 (32‐38)	.098
AMH (ng/mL)	3.9 (2.85‐5.6)	3.65 (2.85‐5.6)	.661
BMI (kg/m^2^)	20.3 (19.1‐21.9)	20.15 (18.875‐22.4)	.789
Total number of COS cycles	1 (1‐2)	1 (1‐2)	.107
FSH at the start of COS (day 3 of menstrual cycle) (mIU/mL)	6.9 (5.8‐8.1)	2.6 (0.7‐7.55)	<.01
LH at the start of COS (day 3 of menstrual cycle) (mIU/mL)	5.4 (4.0‐7.1)	1.4 (0‐3.95)	<.01
Total dose of GnRH antagonist required (mg)	0.75 (0.75‐1.00)	0.75 (0.75‐1.0625)	.438
Total dose of gonadotropin required (IU)	4200.0 (3375‐4875)	4837.5 (4162.5‐5568.75)	<.01
Proportion of patients using GnRHa injection (%) (leuprolide acetate injection)	75.3	63.9	.115
Proportion of patients using GnRHa nasal preparation (%) (intranasal buserelin acetate)	24.7	36.1	
Serum E2 on the day of GnRHa trigger (pg/mL)	15 386 (12339‐18955.5)	13 615.5 (9492.25‐18650.75)	.058
Serum P4 on the day of GnRHa trigger (ng/mL)	3.49 (2.41‐4.88)	3.475 (2.445‐4.6975)	.854
Number of oocytes retrieved after GnRHa trigger	23 (17‐30)	0.5 (0‐1.25)	<.01

Abbreviations: AMH, anti‐Mullerian hormone; COS, controlled ovarian stimulation; E2, estradiol; FSH, follicle‐stimulating hormone; GnRHa, gonadotropin‐releasing hormone agonist; LH, luteinizing hormone; P4, progesterone.

^a^ For the proportions of patients using of each GnRHa medication, comparisons were performed using chi‐square test. Other values are shown as the median (interquartile ranges), and comparisons were performed using the Mann‐Whitney *U* test.

The proportions of patients using each GnRHa medication in the non‐re‐trigger group were 75.3% with injectable leuprolide acetate and 24.7% with intranasal buserelin acetate, whereas those in the re‐trigger group were 63.9% and 36.1%, respectively, with no difference in the choice of GnRHa medication between the groups (chi‐squared test).

### 3.2 Comparisons of patient characteristics and hormone profiles after hCG re‐trigger between the good fertilization group and the poor fertilization group

There were no significant differences in age and AMH level between 17 patients in the good fertilization group and 19 patients in the poor fertilization group (Table 2). The LH and FSH levels at the start of COS (day 3 of the menstrual cycle) were significantly lower in the good fertilization group than in the poor fertilization group (*P* < .01). Total dose of gonadotropin required did not differ between the groups, but the good fertilization group required a significantly smaller dose of GnRH antagonist (*P* < .01). Serum P4 level on the day of hCG re‐trigger was significantly lower in the good fertilization group than in the poor fertilization group (*P* < .01), whereas no significant differences were noted in serum P4 and E2 levels at GnRHa trigger between the groups.

**TABLE 2:** Characteristics and hormone profiles in patients requiring hCG re‐trigger, by the rate of normal fertilization (poor fertilization versus good fertilization group)

	Poor fertilization (rate of normal fertilization <60%)	Good fertilization (rate of normal fertilization ≥ 60%)	*P*‐value^a^
Number of patients	19	17	
*N* (number of cycles)	19	17	—
Age at the start of COS (years)	37 (32.5‐38)	34 (32‐36)	.239
AMH (ng/m)	3.5 (2.45‐4.2)	4.2 (3.1‐7.2)	.168
FSH at the start of COS (day 3 of menstrual cycle) (mIU/mL)	7.1 (2.85‐8.25)	0.7 (0.4‐1.1)	<.01
LH at the start of COS (day 3 of menstrual cycle) (mIU/mL)	3.2 (2.1‐5.4)	0 (0‐0.3)	<.01
Total dose of GnRH antagonist required (mg)	1 (0.75‐1.25)	0.75 (0.5‐1)	<.01
Total dose of gonadotropin required (IU)	4875 (420‐5475)	4800 (4050‐5700)	.937
Proportion of patients using GnRHa injection (%) (leuprolide acetate injection)	68.4	58.8	.549
Proportion of patients using GnRHa nasal preparation (%) (intranasal buserelin acetate)	31.6	41.2	
Serum E2 on the day of GnRHa trigger (pg/mL)	14 218 (8553‐16228)	13 234 (11695‐21325)	.271
Serum P4 on the day of GnRHa trigger (ng/mL)	4 (2.66‐6.46)	3.23 (2.38‐3.8)	.11
Serum P4 on the day of hCG re‐trigger (ng/mL)	12.39 (5.89‐17.11)	3.3 (3.01‐4.6)	<.01
Dose of hCG (IU)	3333 (3166.5‐5000)	3333 (3333‐5000)	.842

Abbreviations: AMH, anti‐Mullerian hormone; COS, controlled ovarian stimulation; E2, estradiol; FSH, follicle‐stimulating hormone; GnRHa, gonadotropin‐releasing hormone agonist; hCG, human chorionic gonadotropin; LH, luteinizing hormone; P4, progesterone.

^a^ For the proportions of patients using each GnRHa medication, comparisons were performed using chi‐square test. Other values are shown as the median (interquartile ranges) and comparisons were performed using the Mann‐Whitney *U* test.

The proportions of patients using each GnRHa medication in the poor fertilization group were 68.4% with injectable leuprorelin acetate and 31.6% with intranasal buserelin acetate, whereas those in the good fertilization group were 58.8% and 41.2%, respectively, with no difference in the choice of GnRHa medication between the groups (chi‐squared test).

### 3.3 Comparisons of clinical outcomes after hCG re‐trigger between the good fertilization group and the poor fertilization group

There were no differences in the numbers of all oocytes and MII oocytes retrieved after hCG re‐trigger between the good fertilization group and the poor fertilization group (Table 3). The good fertilization group had a significantly lower rate of abnormal fertilization, than did the poor fertilization group (*P* < .01). In the good fertilization group, all the patients (17 patients) underwent embryo transfer in or after the next cycle; however, in the poor fertilization group, 9 of the 19 patients (47.4%) had fertilization abnormalities and did not undergo embryo transfer. There were no significant differences in clinical pregnancy rate, clinical loss rate, and live birth rate between the groups.

**TABLE 3:** Clinical outcomes in patients requiring hCG re‐trigger, by the rate of normal fertilization (poor fertilization versus good fertilization group)

	Poor fertilization (rate of normal fertilization <60%)	Good fertilization (rate of normal fertilization ≥ 60%)	*P*‐value^a^
*N* (number of cycles)	19	17	—
Number of total oocytes per retrieval after hCG re‐trigger	11 (7.5‐19.5)	20 (15‐28)	.051
Number of MII oocytes per retrieval after hCG re‐trigger	9 (6‐16.5)	15 (13‐24)	.061
Rate of normal fertilization (2PN) per MII oocyte (%)	20 (10‐30)	80 (70‐80)	<.01
Rate of abnormal fertilization (≥3 PN) per MII oocyte (%)	44.4 (20.8‐55.6)	3.8 (0‐10.5)	<.01
Number of clinical pregnancies/number of patients with embryo transferred (Clinical pregnancy rate)	4/10 (40.0%)	10/17 (58.8%)	.44
Number of patients lost to follow up	1	3	—
Number of patients with available clinical outcome data	3	7	—
Number of clinical losses (rate)	1 (33.3%)	1 (14.3%)	.378
Number of live births (rate)	2 (66.7%)	6 (85.7%)	1.000

Abbreviations: hCG, human chorionic gonadotropin; MII, metaphase II.

^a^ For the number of total and MII oocytes per retrieval and rates of normal and abnormal fertilization, the values are shown as median (interquartile ranges) and comparisons were performed using the Mann‐Whitney *U* test. For clinical pregnancy rate, clinical loss rate, and live birth rate, the values are shown as mean ± SD and comparisons were performed using Fisher's exact test.

### 3.4 Hormone profiles and semen characteristics in male partners

The age, LH, FSH, and total testosterone levels in male partners did not significantly differ between the poor fertilization group and the good fertilization group (Table 4). Semen volume was significantly higher in the good fertilization group than in the poor fertilization group (*P* = .012), but no differences were noted in key factors for successful fertilization by ICSI, such as sperm concentration and sperm motility, between the groups.

**TABLE 4:** Hormone profiles and semen characteristics in male partners (poor fertilization versus good fertilization group)

	Poor fertilization (rate of normal fertilization <60%)	Good fertilization (rate of normal fertilization ≥60%)	*P*‐value^a^
*N* (number of cycles)	19	17	—
Age	36 (33‐40.5)	35 (32‐39)	.382
LH (mIU/mL)	4.5 (3.8‐5.65)	4 (3.4‐5.3)	.333
FSH (mIU/mL)	6.2 (4.05‐7.7)	4.4 (3.1‐6.5)	.136
Total testosterone (ng/mL)	4.67 (3.965‐5.195)	4.77 (3.86‐5.95)	.680
Semen volume (mL)	2.2 (2‐2.95)	3.2 (2.7‐4.2)	.012
Sperm concentration (×10^6^/mL)	27 (6.05‐42.5)	36 (24.7‐43)	.466
Sperm motility (%)	34.2 ± 24.7	47.4 ± 24.5	.119

Abbreviations: FSH, follicle‐stimulating hormone; LH, luteinizing hormone.

^a^ For sperm motility, the values are shown as mean ± SD and comparison was performed using Fisher's exact test. Other values are shown as the median (interquartile ranges), and comparisons were performed using the Mann‐Whitney *U* test.

### 3.5 Morphology of COCs and denuded oocytes

**FIGURE 1:**
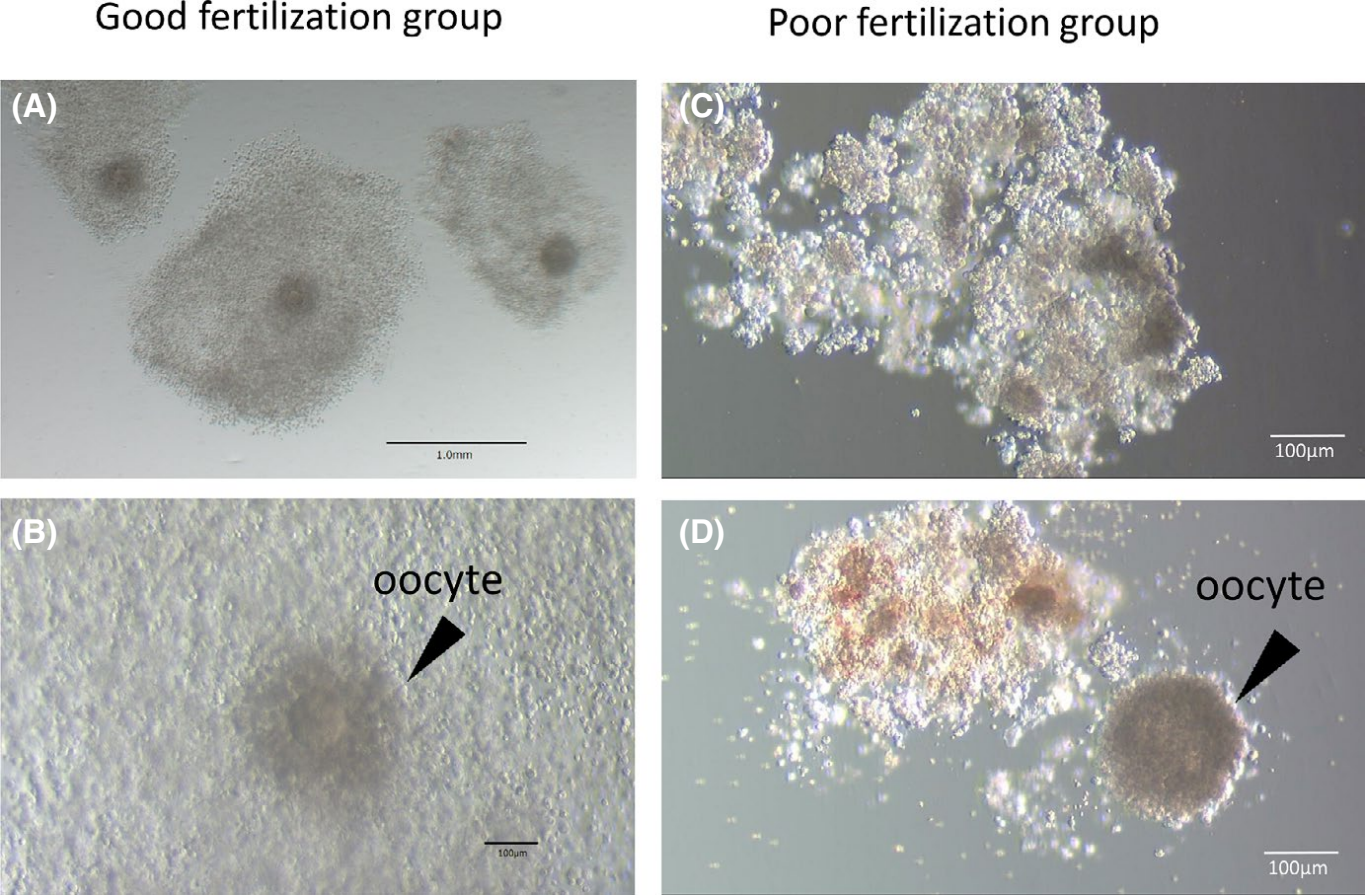
Morphology of cumulus‐oocyte complexes. A, B: Swollen granulosa cells and multiple layers of cumulus cells; C: aggregated granulosa cells; and D: black‐colored granulosa cells

**FIGURE 2:**
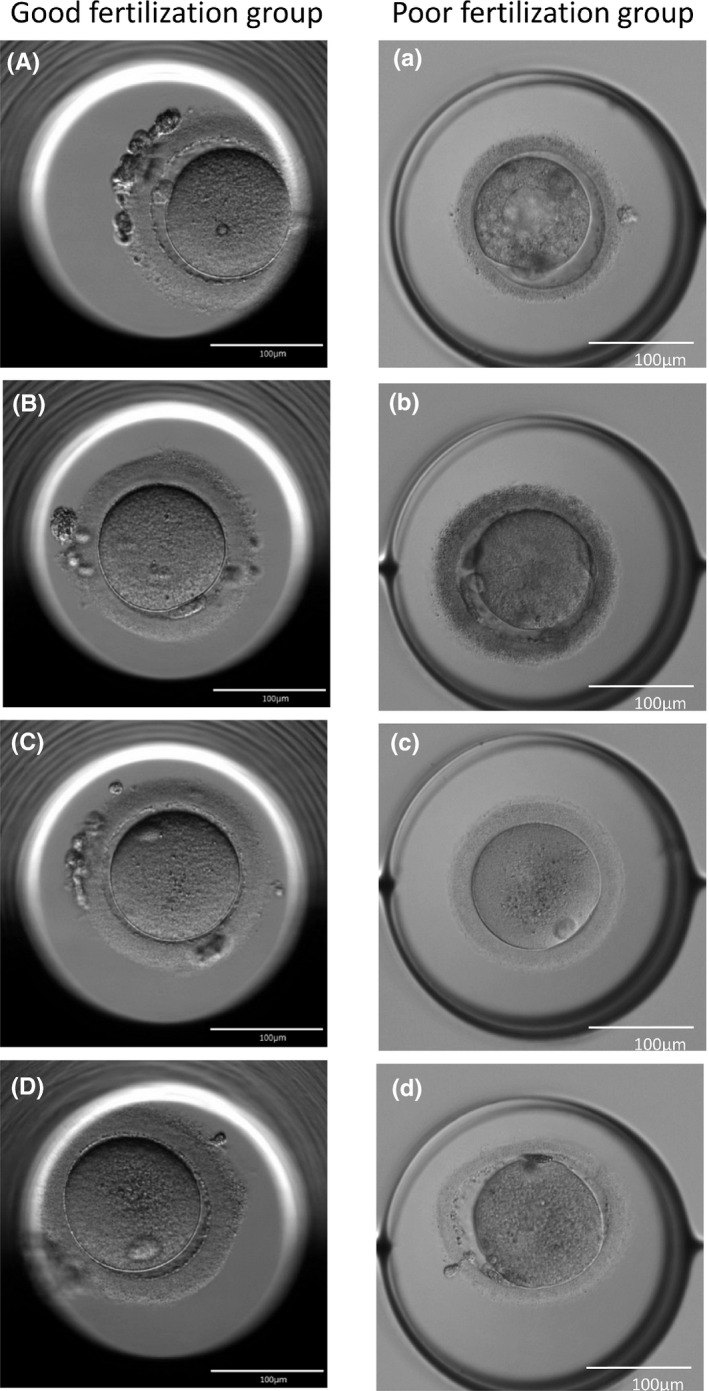
Morphology of denuded oocytes. A, B, C, and D (good fertilization group). No morphological abnormalities were evident. a, b, c, and d (poor fertilization group). a, A huge smooth endoplasmic reticulum (SER) was present in the center; b, the perivitelline space contained abundant debris; c, the oocyte was morphologically normal; and d, the oocyte had rough cytoplasm and fragmented polar bodies

The COCs in the good fertilization group were composed of swollen granulosa cells and multiple layers of cumulus cells (Figure 1A,B). The COCs in the poor fertilization group contained aggregated (Figure 1C) and black‐colored (Figure 1D) granulosa cells. Denuded oocytes, after cumulus cells were removed, in the good fertilization group had no morphologically abnormal findings (Figure 2A‐D). The poor fertilization group, on the other hand, had many denuded oocytes that were deemed morphologically abnormal, though some denuded oocytes were apparently normal (Figure 2c). The morphologically abnormal denuded oocytes observed in the poor fertilization group included oocytes having a large smooth endoplasmic reticulum (SER) in the center (Figure 2a), those with the black‐colored zona pellucida and the perivitelline space containing abundant debris (Figure 2b), and those with rough cytoplasm, slightly oval zona pellucida, and fragmented polar bodies (Figure 2d).

### 3.6 Morphological change over time in fertilized oocytes

**FIGURE 3:**
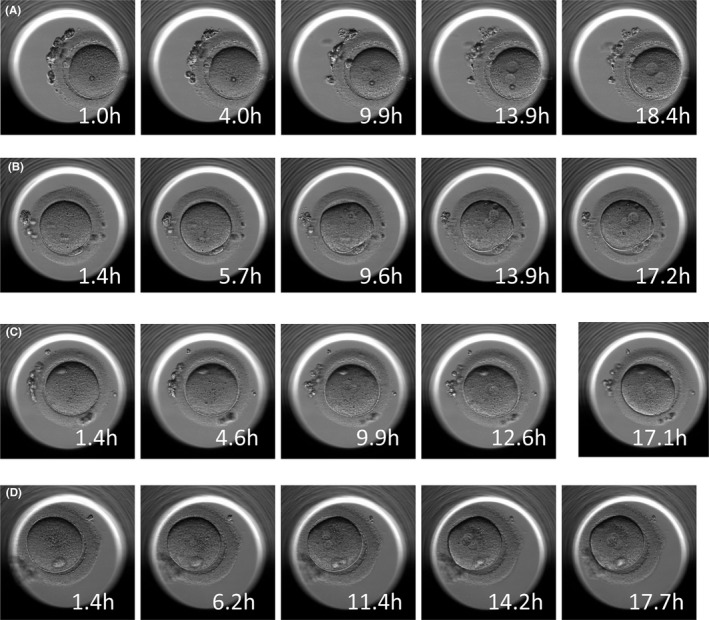
Time‐lapse observation of morphological change over time in oocytes after ICSI in the good fertilization group. A‐C, No morphological change or abnormality observed in the ooplasms. D, poly‐pronuclear embryos occurred during oocyte maturation

**FIGURE 4:**
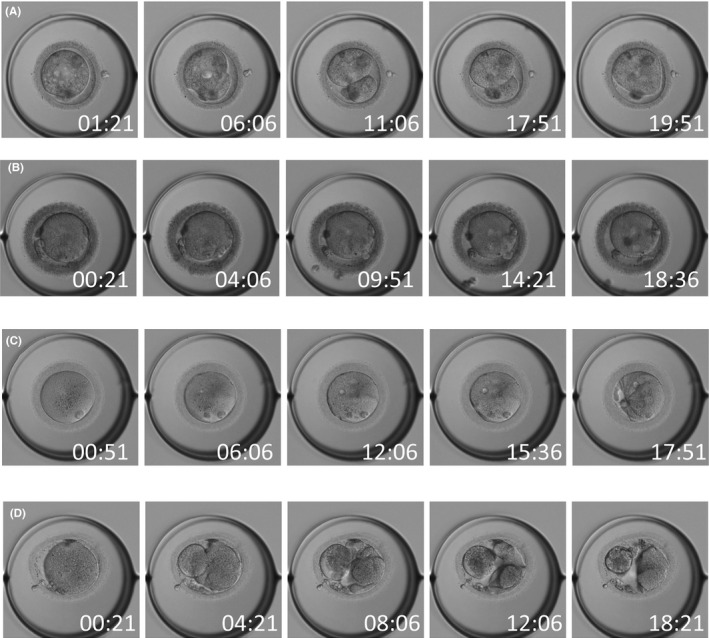
Morphological change over time in oocytes after ICSI in the poor fertilization group. a, Reverse cleavage is shown (oocyte cytoplasm was divided into two before pronuclear formation and subsequently fused back to one); b, normal fertilization is shown, despite debris being evident in the perivitelline space; c, abnormal fertilization is shown resulting from an oocyte that appeared to be morphologically normal before fertilization but had the twisted cytoplasm after fertilization; and d, morphological abnormalities are shown (direct cleavage occurred within several hours after fertilization and multiple distorted and shrunken blastomeres appeared 17.5 h after fertilization.)

Time‐lapse observation of fertilized oocytes in the good fertilization group revealed no morphological change or abnormality in the ooplasm (Figure 3A‐C), except for poly‐pronuclear embryos probably resulting from a failure of polar body extrusion occurring during oocyte maturation (Figure 3D). In the poor fertilization group, on the other hand, marked changes were seen in ooplasmic morphology. Figure 4A shows reverse cleavage, a phenomenon where oocyte cytoplasm was divided into two before pronuclear formation, and subsequently fused back to one. Figure 4B shows normal fertilization, despite debris being evident in the perivitelline space. Figure 4C shows abnormal fertilization resulting from an oocyte that appeared to be morphologically normal before fertilization but had “twisted” cytoplasm after fertilization. Figure 4D shows an oocyte with morphological abnormalities where direct cleavage occurred within several hours after fertilization and multiple distorted and shrunken blastomeres appeared 17.5 hours after fertilization. Thus, abnormal fertilizations occurred through different processes in the good fertilization group and the poor fertilization group.

### 3.7 Cutoff FSH and LH levels at the start of ovarian stimulation (day 3 of the menstrual cycle)

**FIGURE 5:**
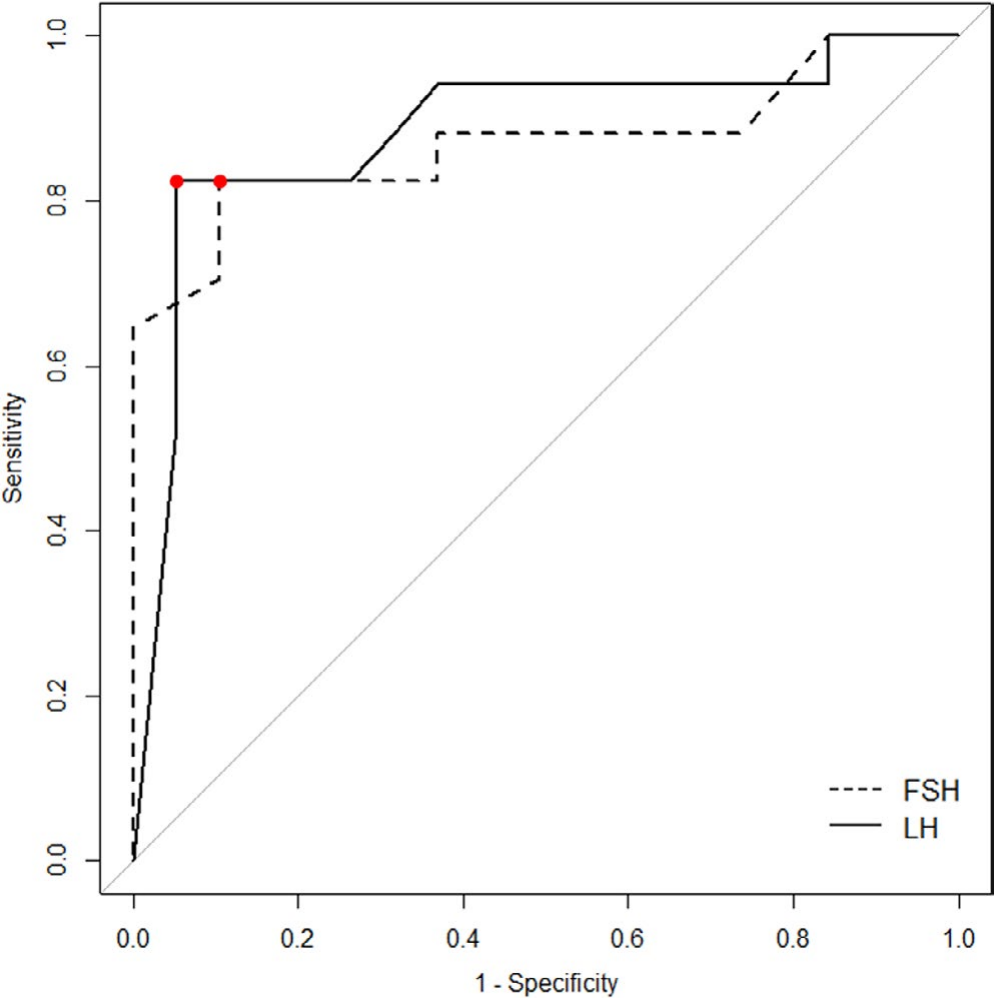
Cutoff FSH and LH levels at the start of ovarian stimulation to predict normal fertilization after re‐trigger. The analysis using receiver operating characteristic showed that an FSH level at the start of COS of 1.30 mIU/mL had a sensitivity of 82.4% and specificity of 89.5% for predicting ≥60% normal fertilization, and that an LH level at the start of COS of 0.35 mIU/mL had a sensitivity of 82.4% and specificity of 94.7% for predicting ≥60% normal fertilization

As above, the good fertilization group had significantly lower FSH and LH levels at the start of COS (both *P* < .01). Based on the results, relationships between FSH and LH levels at the start of COS, and the rate of normal fertilization were assessed using receive operating characteristic (ROC) curves where sensitivity was plotted against 1‐ specificity (Figure 5), and area under curves (AUCs) were calculated to estimate optimal cutoffs of FSH and LH levels at the start of COS to predict normal fertilization after re‐trigger. The AUC values were 0.87 (95% confidence interval [95% CI], 0.74‐1.00) for FSH level and 0.88 (95% CI, 0.79‐1.00) for LH level. The ROC curves also showed that an FSH level at the start of COS of 1.30 mIU/mL had a sensitivity of 82.4% and specificity of 89.5% for predicting ≥60% normal fertilization, and that an LH level at the start of COS of 0.35 mIU/mL had a sensitivity of 82.4% and specificity of 94.7% for predicting ≥60% normal fertilization. Thus, LH level at the start of COS was a more reliable predictor of normal fertilization after re‐trigger than FSH level at the start of COS.

## 4 DISCUSSION

There have been sparse reports as to failures of final oocyte maturation with GnRHa trigger, and the mechanism of the failures has remained unclear.^19^ This is the first report focusing on the endocrine environment at the start of COS and assessing the appropriateness of conducting hCG re‐trigger after failed final oocyte maturation triggered with a GnRHa, based on fertilization outcome.

This study attempted to determine optimal cutoff FSH and LH levels at the start of COS to identify patients requiring re‐trigger in advance. There have been several reports that the need for re‐trigger may be predictable from the endocrine environment after GnRHa trigger. Kummer et al^13^ measured LH and P4 levels at 8 to 12 hours after GnRHa trigger and found that a low LH level of <15 mIU/mL was associated with a suboptimal oocyte yield. Similarly, Shapiro et al reported that a LH level of <12 mIU/mL at 12 hours after GnRHa trigger was associated with a dramatically reduced oocyte yield.^14^

However, measuring hormone levels on the day following GnRHa trigger requires patients to revisit the clinic only for blood sampling; this would increase the burden of patients and may be undesirable in clinical practice. Furthermore, measuring hormone levels on the day following GnRHa trigger in all patients is not practical, in view of the low incidence of failed final oocyte maturation with GnRHa trigger. On the other hand, assessing the endocrine environment at the start of COS, as we propose, may not increase patient's visit frequency.

FSH and LH levels at the start of COS in patients with good fertilization after re‐trigger were significantly lower than those in patients with poor fertilization. Follicular development during COS is endocrinologically regulated by the hypothalamus‐pituitary‐ovary axis. The hypothalamus secretes GnRH in pulses, and in response, the pituitary releases gonadotropins (FSH and LH). Low levels of FSH and LH at the start of COS imply impaired pituitary function. In patients with impaired pituitary function, exogenous FSH administered from day 3 of a menstrual cycle may induce the secretion of E2, which “in turn” inhibits the secretion of endogenous LH and FSH. If a GnRH antagonist is administered under such an endocrine environment, pituitary response would further reduce. In some patients with impaired pituitary function, administration of a GnRHa fails to cause a flare‐up of endogenous FSH and LH, resulting in a suboptimal LH surge, poor oocyte maturity, and inadequate oocyte retrieval. Chang et al^24^ reported that the probability of failed final oocyte maturation with GnRHa trigger was twofold higher in patients with a baseline LH level of 1‐2 mIU/mL than in those with a higher LH level. Meyer et al^15^ found that patients with very low endogenous serum LH levels on the day of LH trigger are at increased risk for a suboptimal GnRHa trigger response, which is generally consistent with our finding. For patients with low FSH and LH levels at the start of COS, hCG trigger may be chosen for final oocyte ovulation. However, the expression of VEGF, which has already begun during ovary stimulation, is increased by hCG administration.^7^, ^25^ VEGF is produced also in luteinized granulosa cells. Administration of hCG in the presence of numerous follicles increases the expression of VEGF,^26^, ^27^ which, in turn, enhances vascular permeability, and thereby inducing OHSS. Thus, the hCG trigger as the first choice should be avoided; however, hCG injection may have to be used for re‐triggering when the GnRHa trigger is not effective like the cases in this study. In such cases, strategies to prevent the development of OHSS may include lower dose of hCG or cabergoline administration. There are various reports regarding the effect of low dose of hCG,^28^, ^29^ and Gunnala et al reported that a single administration of low‐dose hCG (3300 IU, similar to our study) can minimize the incidence of OHSS and facilitate oocyte maturity.^30^ Esinler et al reported that cabergoline administration is a very effective way to reduce the risk of OHSS.^31^ According to the systematic review and meta‐analysis by Leitao et al^32^, cabergoline reduces the occurrence of moderate‐severe OHSS and is unlikely to have a negative impact on clinical pregnancy or on the number of retrieved oocytes. These treatment strategies cannot completely eliminate OHSS but can minimize the incidence of OHSS and control patients' safety of hCG administration.

Although the incidence of abnormal fertilization after ICSI has been reported to be 2.4%‐6.5%,^33^, ^34^, ^35^, ^36^ the rate of abnormal fertilization after hCG re‐trigger in patients with high FSH and LH levels at the start of COS was as high as 44.4% in our study. One possible explanation of the high rate of abnormal fertilization is the in vivo aging of oocytes, which is surmised from in vitro examinations. There have been many reports of oocyte aging. In mice, it was reported that abnormal fertilization increased by postovulatory oocyte aging^37^ and that in vitro aged postovulatory oocytes exhibited chromosomal misalignment and abnormal spindle formation, and failed to reach full‐term development.^38^ It was also reported that human oocytes which had failed to fertilize after an 18‐hour incubation with spermatozoa and had spent a further 6‐8 hours in culture showed an increased incidence of spindle abnormalities and of the proliferation of ooplasmic microtubules,^39^ and chromosomes no longer aligned at the spindle equator but scattered all over the spindles.^40^, ^41^ Zhang et al^42^ reported that the cumulus cells were separated from oocytes after a 24‐hour in vitro incubation of COC. All these previous reports support in vivo oocyte aging.

As mentioned above, low gonadotropin levels imply impaired pituitary function. In patients with low gonadotropin levels, therefore, ovary stimulation with a GnRHa may fail to cause a flare‐up of endogenous FSH and LH, which are affected by pituitary function, resulting in a suboptimal LH surge. For these patients, hCG re‐trigger is effective. After hCG administration, oocytes begin to mature in good conditions, eventually leading to favorable fertilization rates. In patients with high gonadotropin levels, on the other hand, a GnRHa causes a flare‐up of endogenous FSH and LH and induces an optimal LH surge, in the presence of normal pituitary function. In the poor fertilization group, patients had high P4 levels at hCG re‐trigger, suggestive of the occurrence of luteinization. This indicates that oocyte maturation had already begun with first trigger with a GnRHa. The necessity of re‐trigger is judged solely based on difficulty to collect oocytes. During the 2 days, until the second oocyte retrieval was conducted, oocytes may have aged and overmatured in the body, leading to the dispersion of chromosomes on spindles and the increased incidence of abnormal fertilization. In patients with high gonadotropin levels at the start of COS, a certain number of oocytes may be collected by continuing the first oocyte aspiration attempt, without putting additional burdens of re‐trigger and subsequent oocyte respiration on patients.

Failures of final oocyte maturation triggered with a GnRHa requiring hCG re‐trigger occur with an extremely low frequency, and the endocrine environment can fluctuate even within the same patient. It can be therefore said that low FSH and LH levels at the start of COS are not direct causes of infertility but may be incidental symptoms. There have been many reports regarding suboptimal oocyte yield after GnRHa trigger^43^; however, most of the reports describe the possible association of hormone levels with oocyte yield after re‐trigger, but not with fertilization or embryo development. Further investigation will be necessary focusing on the association of hormone levels with fertilization, embryo development, pregnancy, and delivery after re‐trigger.

Currently, there are no established criteria or protocols to determine the appropriateness of hCG re‐trigger. This study proposes that a FSH level of ≤1.30 mIU/mL and a LH level of ≤0.35 mIU/mL at the start of COS are expected to be optimal predictors of poor pituitary response to GnRHa trigger and need for hCG re‐trigger. Conducting re‐trigger solely based on the fact that adequate oocytes were not retrieved by the first oocyte aspiration attempt may cause oocyte overmaturation resulting in the increased incidence of abnormal fertilization. Low levels of FSH and LH at the start of COS, in combination with the fact that adequate number of oocytes were not retrieved, would warrant the conduct of hCG re‐trigger to collect normally fertilized oocytes. Gonadotropin levels, that is, a FSH level of ≤1.30 mIU/mL and a LH level of ≤0.35 mIU/mL at the start of COS are predictors of poor pituitary response to GnRHa trigger and the necessity of hCG re‐trigger, and may serve as indicators to help clinicians appropriately choose hCG re‐trigger rather than abandoning the cycles or continuing the first oocyte aspiration attempt.

## DISCLOSURES

*Conflict of interest*: The authors declare no conflict of interest. *Human rights statements and informed consent*: All the procedures were followed in accordance with the ethical standards of the responsible committees on human experimentation (institutional and national) and with the principles of the Helsinki Declaration of 1964 and its later amendments. This study was approved by the Institutional Review Board of Asada Ladies Clinic. This is a retrospective study in patients who submitted informed consent for undergoing fertility treatment at our IVF center. *Animal studies:* This article does not contain any study with animal participants that have been performed by any of the authors.
